# Supplementary material for: Anti-SARS-CoV-2 Activity of Extracellular Vesicle Inhibitors: Screening, Validation, and Combination with Remdesivir
Source: Biomedicines. 2021 Sep 16;9(9):1230. doi: 10.3390/biomedicines9091230 (PMC8465755; doi:10.3390/biomedicines9091230)
Supplement: Supplementary file 1 [file biomedicines-09-01230-s001.zip › Supplementary materials/Figure S1_R1.pdf]

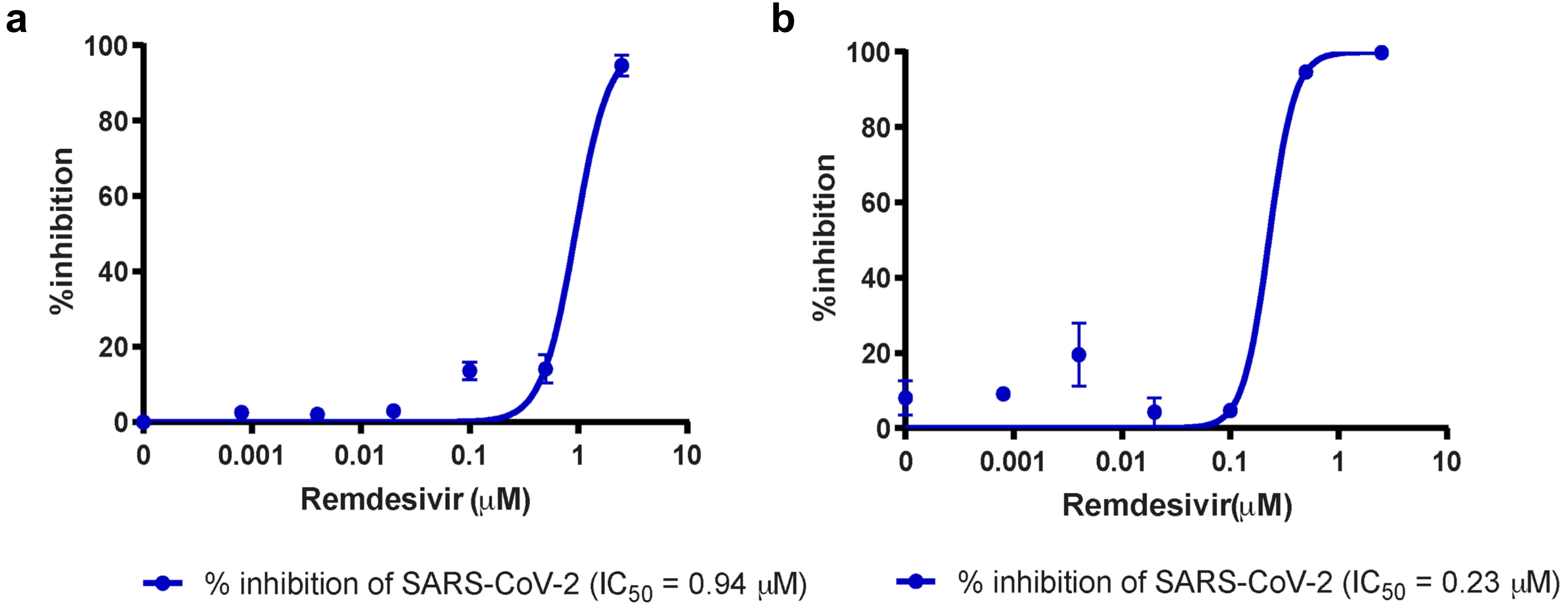

Figure S1: The IC50 and CC50 of remdesivir in Vero E6 (a) and Calu-3 (b) cells. Vero E6 and Calu-3 cells were infected with SARS-CoV-2 at 25TCID50 for 2h and then post-infection treated with calpeptin at concentrations ranging from 0.0008 μM to 2.5 μM for 48h. The infected cells were fixed and stained for viral nucleoproteins with anti-SARS-CoV NP mAb. The SARS-CoV-2 infected cells were detected by high-content imaging. The percentage of inhibition of infected cell was calculated as percentage of the control conditions. The data are presented as the mean ± SEM of three biological replicates
